# *Vibrio splendidus* Fur regulates virulence gene expression, swarming motility, and biofilm formation, affecting its pathogenicity in *Apostichopus japonicus*

**DOI:** 10.3389/fvets.2023.1207831

**Published:** 2023-06-05

**Authors:** Yue Shi, Changyu Liao, Fa Dai, Yiwei Zhang, Chenghua Li, Weikang Liang

**Affiliations:** ^1^State Key Laboratory for Managing Biotic and Chemical Threats to the Quality and Safety of Agro-Products, Ningbo University, Ningbo, Zhejiang, China; ^2^Laboratory for Marine Fisheries Science and Food Production Processes, Qingdao National Laboratory for Marine Science and Technology, Qingdao, China

**Keywords:** *Vibrio splendidus*, *Apostichopus japonicus*, ferric uptake regulator, biofilm, swarming motility

## Abstract

*Vibrio splendidus* is an opportunistic pathogen that causes skin ulcer syndrome and results in huge losses to the *Apostichopus japonicus* breeding industry. Ferric uptake regulator (Fur) is a global transcription factor that affects varieties of virulence-related functions in pathogenic bacteria. However, the role of the *V. splendidus fur* (*Vsfur*) gene in the pathogenesis of *V. splendidus* remains unclear. Hence, we constructed a *Vsfur* knock-down mutant of the *V. splendidus* strain (MTVs) to investigate the role of the gene in the effect of biofilm, swarming motility, and virulence on *A. japonicus*. The result showed that the growth curves of the wild-type *V. splendidus* strain (WTVs) and MTVs were almost consistent. Compared with WTVs, the significant increases in the transcription of the virulence-related gene *Vshppd* mRNA were 3.54- and 7.33-fold in MTVs at the OD_600_ of 1.0 and 1.5, respectively. Similarly, compared with WTVs, the significant increases in the transcription of *Vsm* mRNA were 2.10- and 15.92-fold in MTVs at the OD_600_ of 1.0 and 1.5, respectively. On the contrary, the mRNA level of the flagellum assembly gene *Vsflic* was downregulated 0.56-fold in MTVs at the OD_600_ of 1.0 compared with the WTVs. MTVs caused delayed disease onset time and reduced *A. japonicus* mortality. The median lethal doses of WTVs and MTVs were 9.116 × 10^6^ and 1.658 × 10^11^ CFU·ml^−1^, respectively. Compared with WTVs, the colonization abilities of MTVs to the muscle, intestine, tentacle, and coelomic fluid of *A. japonicus* were significantly reduced. Correspondingly, the swarming motility and biofilm formation in normal and iron-replete conditions were remarkably decreased compared with those of WTVs. Overall, these results demonstrate that Vsfur contributes to the pathogenesis of *V. splendidus* by regulating virulence-related gene expression and affecting its swarming and biofilm formation abilities.

## Introduction

The gram-negative rod-shaped bacterium *Vibrio splendidus* can cause severe vibriosis in numerous aquatic animals (1, 2). In particular, it is the main pathogen of “skin ulcer syndrome” (SUS), which occurs in sea cucumbers, *Apostichopus japonicus* (Echinodermata, Holothuroidea) (3, 4). Sea cucumber farming frequently suffers from SUS, resulting in serious economic losses (5). In recent years, research on the immune mechanism of sea cucumbers has made great progress. Different pattern recognition receptors that recognize pathogens have been identified (6, 7), complex immune regulatory networks have been depicted, and the relationship between metabolism and immunity has been further explored (8). Unfortunately, there is still a lack of effective means to prevent SUS. The fundamental reason is that research on the pathogenic mechanism is relatively scarce. It mainly focuses on the exploration of *V. splendidus* virulence factors such as hemolysin and extracellular metalloprotease (9, 10), lacking the cross integration of host immunity, pathogenic pathogenicity, and environmental factors.

Environmental factors have an important impact on the virulence and survival of pathogenic bacteria. One important environmental signal is iron, which is an essential micro-nutrient in almost all living organisms (11). However, excessive intracellular iron concentration is toxic to bacteria due to the reactive oxygen species produced by Fenton and Haber-Weiss reactions (12, 13). Therefore, many pathogens must control the iron concentration within a certain range (14). Ferric uptake regulator (Fur), a global transcription regulator factor, is a key regulator for bacteria to respond to changing iron availability in the environment, thereby regulating bacterial gene expression related to iron uptake, utilization, and storage (15, 16). Significantly, the *fur* gene also plays a critical role in the expression of virulence-related genes, such as oxidative stress response, quorum sensing, swarming motility, and biofilm formation in numerous pathogens during host invasion (17–20). Under normal and iron-replete conditions, Fur binds to Fe^2+^ as an inhibitor and then combines with the promoter region. It inhibits the transcription of the target gene by preventing RNA polymerase from binding to the target gene. The typical representative of this regulation mode is the siderophore transportation system (21, 22). Under iron-poor conditions, Fur combines with a conserved DNA sequence called the “*fur* box,” which is located near promoter regions and subsequently activates or inhibits transcription of the target genes (23). Moreover, the regulation of Fur can occur indirectly via small RNAs (24).

Our previous study confirmed that the iron uptake pathway of *V. splendidus* depends on hydroxamate siderophore-IutA (25). Alignment of the amino acid sequence demonstrated that *V. splendidus fur* (Vsfur) showed high similarities to that of the Furs from other *Vibrio* sp. (26). However, the regulation of bacterial pathogenicity caused by Vsfur is not known. In the present study, the *Vsfur* gene was knocked down by antisense RNA interference. The *Vsfur* knock-down mutant of *V. splendidus* (MTVs) was assessed for growth curve and expression of virulence-related genes compared with wild-type *V. splendidus* (WTVs). The WTVs and MTVs cells were collected to analyze swarming motility and biofilm formation in normal, iron-replete, and iron-starved conditions. Furthermore, the colonization ability and pathogenicity of WTVs and MTVs in *A. japonicus* were analyzed and compared.

## Materials and methods

### Bacterial strains, culture conditions, and chemicals

The wild-type *V. splendidus* strain was dissociated from the focus of sea cucumbers with SUS and preserved in our laboratory. Zhang et al. tested the strain’s pathogenicity in sea cucumbers by means of a reinfection experiment (27). The bacterial cultures of the wild-type strain and indicated mutant were shaken (180 rpm) at 28°C in Zobell’s 2216E medium (5 g·L^−1^ tryptone, 1 g·L^−1^ yeast extract in filtered natural seawater). *Escherichia coli* S17λ*pir* and DH5α (Takara, China) were grown in Luria–Bertani (LB) broth or agar at 37°C. The antimicrobial agent was purchased from a commercial source Sangon (Shanghai, China). Antibiotics were added to the medium at the following concentrations: kanamycin (Kn) 50 μg·ml^−1^, gentamicin (Gm) 100 μg·ml^−1^, and ampicillin (Ap) 100 μg·ml^−1^. The vectors used in the experiment were pMD19-T, which was purchased from Takara (Beijing, China); vector pBBR1MCS-5, which was purchased from Fenghui Biotechnology (Changsha, China); and vectors pET28a and pBT3, which were kept in our laboratory. Restriction endonucleases were purchased from New England Biolabs and used according to the manufacturer’s instructions.

### Mutant of *Vibrio splendidus* construction

The primers used in the present study were designed according to the genomic DNA of the *V. splendidus* strain LGP32 with accession number FM954973.2 and listed in Table 1. MTVs was constructed as described previously (28, 29). Plasmid pET28aVsfur containing constitutive promoter P_Trc_ was generated to construct the MTVs with the reverse *Vsfur* sequence. The antisense RNA fragment of *Vsfur* was amplified with the forward primer VsfurF (*Xho* I) and reverse primer VsfurR (*Bam*H I) and ligated into pMD19-T for subsequent restriction digestion. Plasmid pBT3Vsfur was constructed by ligating *Vsfur* into pBT3, between the *Bam*H I/*Xho* I sites. The reverse *Vsfur* fragment fusing Trc promoter was obtained after pBT3Vsfur was digested with *Swa* I. The P_Trc_-*Vsfur* fragment was inserted into the blunt end site *Eco*R V of pET28a and then transformed into S17λπ to construct S17λπ/pET28aVsfur. To transform the recombinant plasmid pET28aVsfur into wild-type *V. splendidus*, bacterial conjugation was conducted as described previously (30). The conjugants were placed on 2216E agar together with Kn and Ap, and the positive conjugants were picked out and confirmed by PCR (T7/T7ter) and sequencing.

**Table 1:** Primers used in this study.

Primer	Sequences (5′–3′)^a^
VsfurF	CTCGAGATGTCAGACAATAATCAAGCG (*Xho* I)
VsfurR	GGATCCAGTTACAATGCCAGCATC (*Bam*H I)
fur^C^F	GGATCCATCTTCTCTGAATTGAGGCTTCTTTC (*Bam*H I)
fur^C^R	CTCGAGTTATTTCTTCGCTTTGTGTGCGTCT (*Xho* I)
T7	TAATACGACTCACTATAGGG
T7ter	GCTAGTTATTGCTCAGCGG
pBBR1MCS-5F	CAGGAAACAGCTATGACC
pBBR1MCS-5R	TGTAAAACGACGGCCAGT
8F	AGAGTTTGATCCTGGCTCAG
1492R	GGTTACCTTGTTACGACTT
VsfurRTF	TCACCACGATCACCTAGTATGTTT
VsfurRTR	GCAATCCCCAGTGATGCTTTT
VshppdRTF	GCCAAGCACCGTTCAAAAGA
VshppdRTR	GAAAAGCCATGCCACACACC
VsmRTF	CTCCAACAGAGCCTCGTCG
VsmRTR	GTTCTCATCCAATCTCACCATCA
VsflicRTF	TGTGACCGATGTGGGTGGAG
VsflicRTR	CATTTGAGTAGTTTCTTTGGCGTAG
933F	GCACAAGCGGTGGAGCATGTGG
16SRTR1	CGTGTGTAGCCCTGGTCGTA

^a^Underlined nucleotides are the restriction sites of the enzymes indicated in brackets at the end of the sequences.

### Complementation of the fur deletion mutant of *Vibrio splendidus*

To complement the *fur* deletion mutant of *V. splendidus*, we designed a pair of primers, fur^C^F/R, to amplify the promoter region and complete ORF of the *Vsfur* gene. The fragment was ligated into pMD19-T and digested with *Bam*H I and *Xho* I. Subsequently, fragments with *Bam*H I and *Xho* I cleavage sites were cloned into the wide-host vector pBBR1MCS-5, which had different replicon and antibiotic resistance from pET28a. The recombinant vector pBBR1MCS-5Vsfur was transferred into MTVs by bacterial conjugation. The positive conjugants were selected on 2216E agar plates amended with Kn and Gm. Finally, the complemented strain *fur^C^* was confirmed by PCR (pBBR1MCS-5F/R) and used for further research.

### Growth curve measurement

*Vibrio splendidus* strains, including WTVs, MTVs and *fur^C^* were coated to 2216E agar at 28°C for 24 h. Single colonies were transferred into 10 ml of fresh 2216E broth and allowed to reach the optical density at 600 nm (OD_600_) of 1.0. The culture was diluted proportionally to the same concentration. One hundred microlitre aliquots of WTVs, MTVs, and *fur^C^* were reinoculated into flasks with 100 ml of fresh 2216E broth, 2216E broth supplemented with 100 μM iron chelator 2,2′-dipyridyl (DP), and 2216E broth supplemented with 50 μM FeCl_3_ and grown at 28°C with agitation at 180 rpm. The OD_600_ was recorded at different time points with an ultraviolet spectrophotometer (Mapada Instruments Co. Ltd., Shanghai, China). The experiment was repeated three times for each sample.

### Quantification of mRNA expression

Total RNA was isolated from WTVs and MTVs cells at different OD_600_ values. Data were standardized with the endogenous reference gene 16S rDNA (933F and 16SRTR1). The PrimeScript RT kit (Takara, Japan) and the reagent for removing genomic DNA were used for reverse transcription. Real-time polymerase chain reaction (RT-PCR) was performed in an ABI 7500 RT-PCR detection system (Applied Biosystems) with a total volume of 20 μl containing 0.4 μl of Dye-II (ROX), 0.8 μl of each of the forward and reverse primers (10 μM), 8 μl of diluted cDNA template, and 10 μl of 2 × SYBR Green Mix. The experimental procedure was set as follows: 95°C for 5 min to activate the polymerase, 40 cycles of 95°C for 15 s, 60°C for 20 s, and 72°C for 20 s. Fluorescent signals were collected at the extension stage of each cycle. The primers for RT-PCR are listed in Table 1. The fold changes of gene expression were determined using the 2^−ΔΔCT^ method (31).

### Artificial infection

The sea cucumber artificial infection experiments proceeded as described by Liang et al. (32). Healthy sea cucumbers (weight, 3 ± 1 g) were temporarily reared for 2–3 days before the experiment. Then, 105 sea cucumbers were randomly divided into seven groups. Each group was placed in an aquarium containing 10 ml of aerated natural seawater (salinity, 28 psu), and the temperature was maintained at approximately 16°C. For microbial infection experiments, the WTVs and MTVs strains were prepared as described in culture conditions. The cells were collected when OD_600_ reached 1.0 by centrifugation at 8000 × *g,* washed three times with PBS, and resuspended in seawater. Three groups were continuously immersed in 1 × 10^7^, 1 × 10^6^, and 1 × 10^5^ CFU·ml^−1^ WTVs and the other three groups were immersed in 1 × 10^7^, 1 × 10^6^, and 1 × 10^5^ CFU·ml^−1^ MTVs. At the same time, the negative control group was inoculated with sterile PBS. The symptoms of infected *A. japonicus* were observed and the daily mortality was recorded.

### Colonization quantities of *Vibrio splendidus* in *Apostichopus japonicus* tissues

The distributions of *V. splendidus* in the different *A. japonicus* tissues were determined as described previously (32). Sea cucumbers in the same growth state were equally divided into two groups, and each group was infected by WTVs and MTVs in a final concentration of 10^7^ CFU·ml^−1^. After *A. japonicus* was subjected to immersion infection for 48 h, the body wall, muscle, respiratory tree, intestine, and tentacle of *A. japonicus* were collected using sterilized scissors and tweezers. Three tissue samples from three sea cucumbers were collected and mixed together; this was repeated three times. The specimens were supplemented with sterile PBS to 2 ml. Subsequently, the mixtures were homogenized by a homogenizer. One hundred microlitre gradient diluted mixtures were spread on 2216E agar plates supplemented with Ap (100 μg·ml^−1^). Meanwhile, the coelomic fluids were filtered through a 200-mesh and spread on 2216E agar plates supplemented with Ap. Single colonies from each plate were identified by 16S rDNA sequencing analysis and the number of colonies was counted after incubation at 28°C for 24 h.

### Motility analysis

Swarming motility was assayed as described previously (33). WTVs, MTVs, and *fur^C^* strains were separately grown in fresh 2216E broth at 28°C with agitation at 180 rpm, and OD_600_ was adjusted to 1.0. Then, 2 μl bacterial cell suspension was dropped on the center of 2216E, 2216E supplemented with 200 μM iron chelator DP, and 2216E supplemented with 100 μM FeCl_3_ plates containing 0.3% agar. The 2216E agar plates were then incubated at 28°C for 3 days to observe the diameters of the swarming halos. The iron treatment with different concentrations was repeated three times.

### Biofilm formation assay

Biofilm formation assays for WTVs, MTVs, and *fur^C^* were conducted as described by Luo et al. (34). Briefly, the WTVs, MTVs, and *fur^C^* strains were prepared as described in culture conditions. The cell suspensions were adjusted to OD_600_ = 1.0 with fresh 2216E broth. Then, 2 μl of WTVs, MTVs, and *fur^C^* strains were transferred into 198 μl of 2216E broth, 2216E broth supplemented with 200 μM DP, and 2216E broth supplemented with 100 μM FeCl_3_ in a 96-well plate and allowed to grow at 28°C for 48 h. Subsequently, the plate was washed three times with sterile PBS. Biofilms were stained with 200 μl of crystal violet (1%) for 30 min and washed with sterile PBS. The culture plate was then placed upside down on the absorbent paper. The adsorbed biofilm was dissolved with 200 μl of ethanol and the OD_570_ was measured. The iron treatment with different concentrations was conducted in five independent biological replicates.

### Statistical analyses

Promoter prediction analyses were conducted with online prediction tools at http://www.softberry.com. The 500-bp upstream sequences of virulence-related genes from the initial codon site were used to search the Fur binding box. All data are expressed as the mean ± SD of at least three sets of independent experiments. Statistical significance was determined by one-way Analysis of Variance (ANOVA) with a Dunnett’s test. The significance level was defined as *p < 0.05, **p < 0.01.

## Results

### The role of *Vsfur in vitro Vibrio splendidus* growth

**Figure 1:**
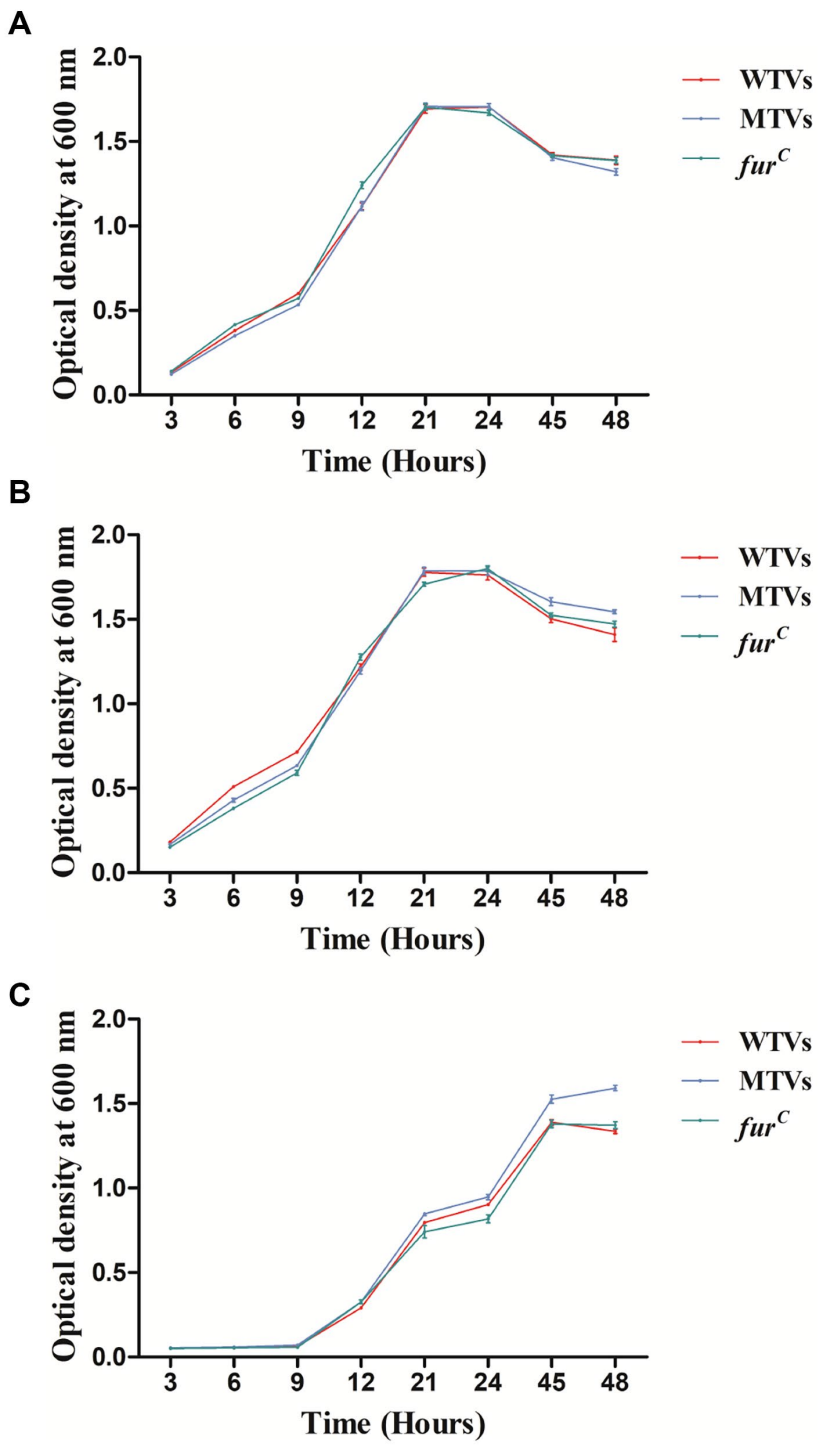
Growth curves of WTVs, MTVs, and *fur^C^*. WTVs, MTVs, and *fur^C^* were spread onto 2216E solid plates at 28°C overnight. Three single colonies were inoculated into flasks with 100 ml of fresh 2216E medium and incubated at 28°C with shaking at 180 rpm. Overnight cultures were diluted to the same concentration, and 200 μl aliquots of WTVs, MTVs, and *fur^C^* were transferred into flasks with 100 ml of fresh 2216E broth **(A)**, 2216E broth supplemented with 50 μM FeCl_3_ **(B)**, and 2216E broth supplemented with 100 μM iron chelator DP **(C)**. OD_600_ values were measured at different time points.

**Figure 2:**
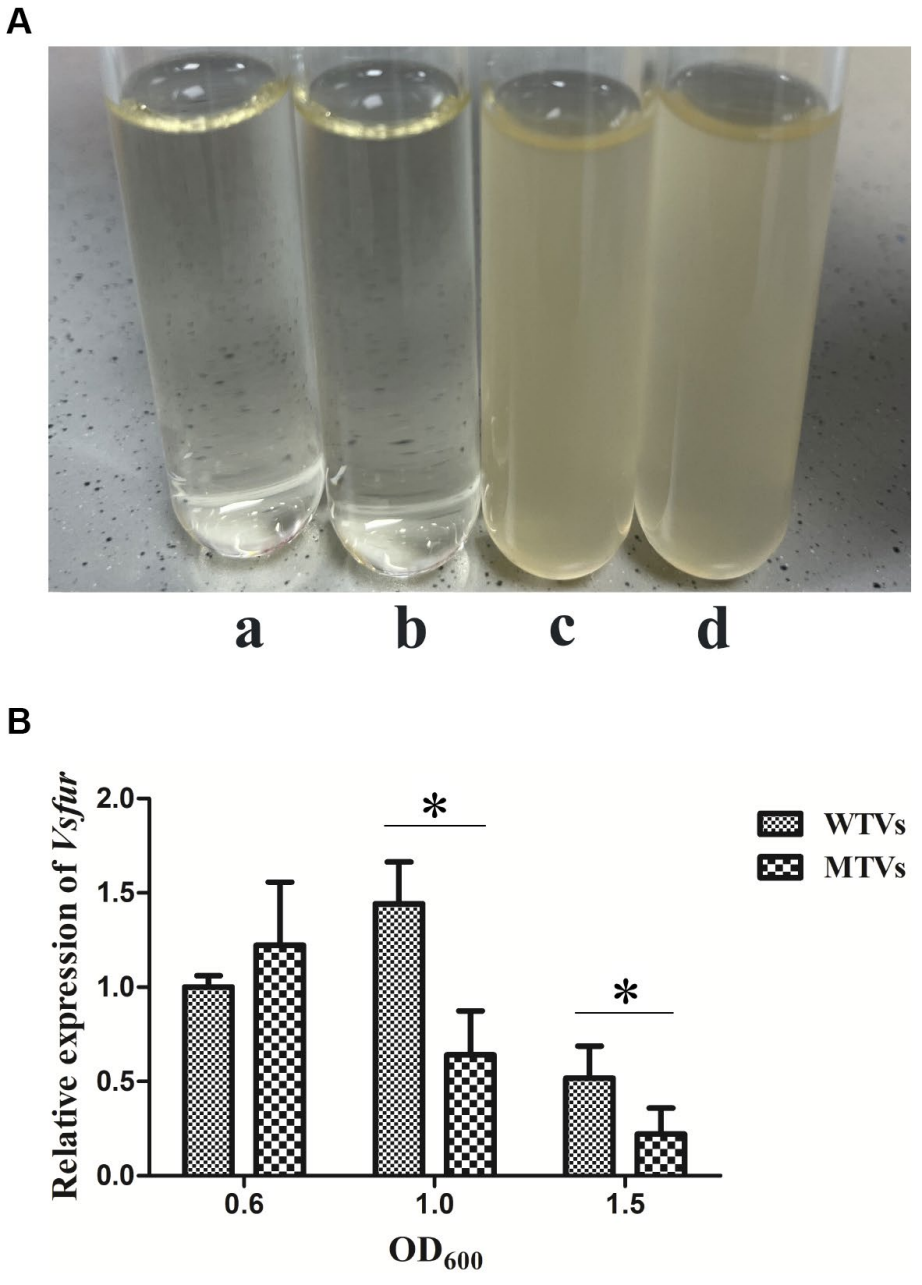
**(A)** Antibiotic resistance test of WTVs, MTVs, and *fur^C^*. WTVs and MTVs were spread onto 2216E solid plates at 28°C overnight. Single colonies were inoculated into tubes with 5 ml of fresh 2216E medium supplemented with Ap and Kn and incubated at 28°C with shaking at 180 rpm. **(a)** Negative control group, **(b)** WTVs group, and **(c)** MTVs group. *fur^C^* was spread onto 2216E solid plates at 28°C overnight. Single colonies were inoculated into tubes with 5 ml of fresh 2216E medium supplemented with Ap, Kn and Gm and incubated at 28°C with shaking at 180 rpm. **(d)** *fur^C^* group. **(B)** Temporal expression analyses of *Vsfur* in WTVs or MTVs at the OD_600_ of 0.6, 1.0, and 1.5. Values are presented as mean ± SD (*n* = 5). Asterisks indicate significant differences: *p < 0.05 and **p < 0.01.

The growth curves of WTVs, MTVs, and *fur^C^* were measured under different iron concentration culture conditions. The OD_600_ was measured at 3, 6, 9, 12, 21, 24, 45, and 48 h. Although iron chelator DP inhibited the growth of three strains within 24 h, the growth curves of the WTVs, MTVs, and *fur^C^* strains were almost the same in 2216E broth (Figure 1A), 2216E broth supplemented with 50 μM FeCl_3_ (Figure 1B), and 2216E broth supplemented with 100 μM DP (Figure 1C). In this study, *Vsfur* was knocked down by antisense RNA interference to construct MTVs. Our previous research demonstrated that *V. splendidus* showed resistance to ampicillin, which is a *β*-lactam antibiotic, but *V. splendidus* was sensitive to kanamycin (35). Thus, plasmid pET28a with kanamycin resistance, which can replicate in *V. splendidus*, was used as a carrier vector to introduce an exogenous DNA fragment containing P_Trc_-*Vsfur*. Meanwhile, constitutive promoter P_Trc_ could regulate the mRNA expression constant to a certain extent, improving the interference efficiency. Single colonies of WTVs and MTVs were inoculated into tubes with 10 ml of fresh 2216E medium supplemented with Ap and Kn cultured at 28°C overnight. No bacteria grew in the blank control and WTVs group, and the bacteria of MTVs could grow in the 2216E medium supplemented with Ap and Kn (Figure 2A). These results suggest that the recombinant plasmid pET28aVsfur was successfully transformed into *V. splendidus* (MTVs) and had no obvious influence on the growth rate of *V. splendidus*.

### The differential expression of virulence-related genes between WTVs and MTVs

**Figure 3:**
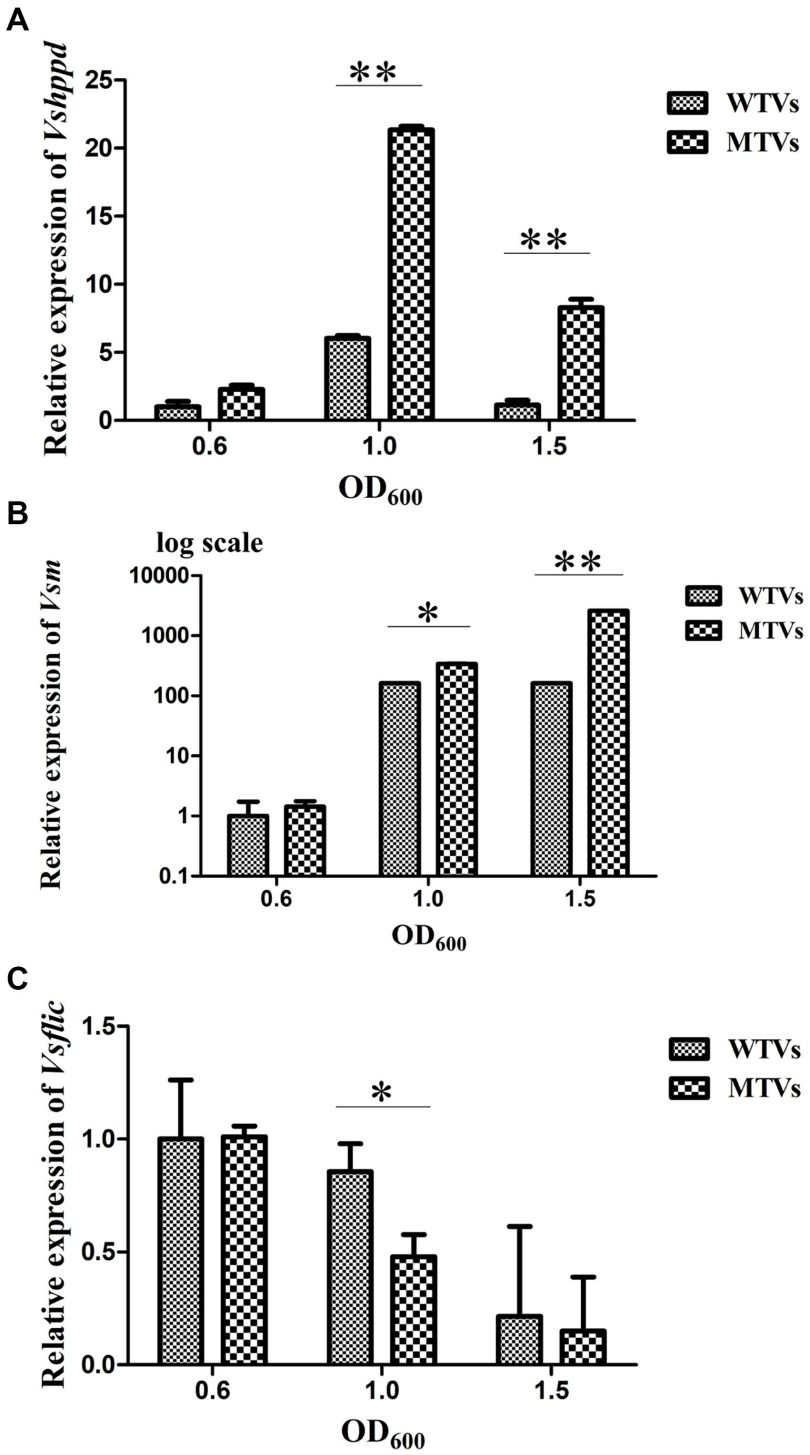
Temporal expression analyses of **(A)** *Vshppd*, **(B)** *Vsm* (The *Y*-axes were shown as a log scale), and **(C)** *Vsflic* in WTVs or MTVs at the OD_600_ of 0.6, 1.0, and 1.5. Values are presented as mean ± SD (*n* = 5). Asterisks indicate significant differences: *p < 0.05 and **p < 0.01.

The WTVs and MTVs cells were collected at the OD_600_ of 0.6, 1.0, and 1.5 to determine the temporal expressions of *Vsfur* and virulence-related genes. The expression levels of *Vsfur*, *Vshppd*, *Vsm,* and *Vsflic* from MTVs were not significantly affected in the early log phase at the OD_600_ of 0.6. The expression of the *Vsfur* mRNA level was significantly downregulated 0.44- and 0.43-fold (*p* < 0.05) in MTVs at the OD_600_ of 1.0 and 1.5 compared with the WTVs (Figure 2B). Compared with the WTVs, the significant increases in the transcription of *Vshppd* mRNA were 3.54- and 7.33-fold (*p* < 0.01) in MTVs at the OD_600_ of 1.0 and 1.5 (Figure 3A). Meanwhile, the expression of *Vsm* mRNA was extremely significantly upregulated at the OD_600_ of 1.0 and 1.5 compared with the OD_600_ of 0.6. Compared with the WTVs, the significant increases in the transcription of *Vsm* mRNA were 2.10- and 15.92-fold (*p* < 0.01) in MTVs at the OD_600_ of 1.0 and 1.5 (Figure 3B). Under the same conditions, the mRNA level of *Vsflic* was downregulated 0.56-fold (*p* < 0.05) in MTVs at the OD_600_ of 1.0 compared with the WTVs (Figure 3C). These results suggest that the expression of the *Vsfur* gene was knocked down successfully in the mid and stationary log phase. Moreover, the downregulation of *Vsfur*, a global transcription factor that affects a number of virulence-related genes in *V. splendidus*, including upregulated genes in the MTVs for iron acquisition *Vshppd* (hemolysin) and *Vsm* (metalloprotease) and downregulated genes such as *Vsflic* (flagella C) related to flagellum biosynthesis. In summary, *Vsfur* has both positive and negative regulatory functions.

### The promoter regions from the functional genes showed Fur binding sites

**Figure 4:**
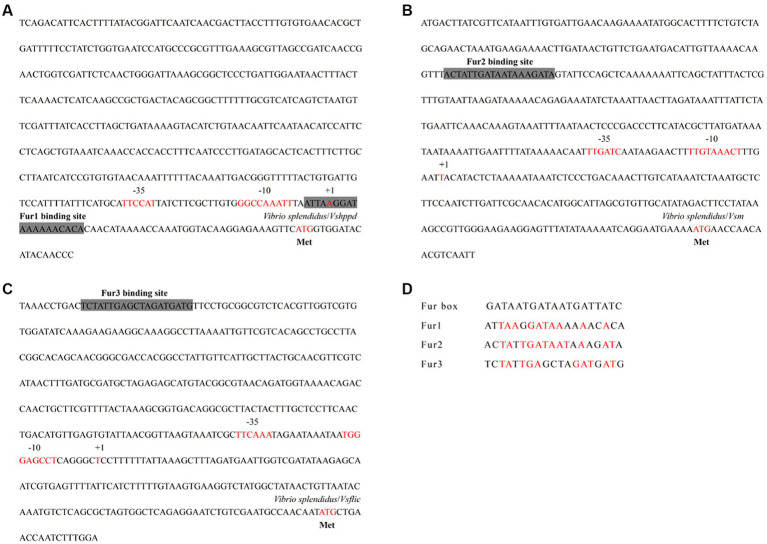
The analysis for Fur binding box prediction in the **(A)** *Vshppd*, **(B)** *Vsm,* and **(C)** *Vsflic* promoter region. The putative Fur binding boxes are indicated as gray boxes. The transcriptional initiation site, the corresponding −10 and −35 boxes, and the translational start site are indicated in red letters. **(D)** Sequence alignment of these putative Fur binding boxes with the Fur box consensus sequence. Bases identical to the consensus are shown in red.

Promoter sequence analyses from the virulence-related genes of *Vshppd*, *Vsm,* and *Vsflic* were performed to predict the putative Fur binding boxes in the *V. splendidus* strain. The promoter regions before the ATG initial cordon of the three virulence-related genes were searched in the genomic DNA database. BPROM^1^ prediction proved that the upstream of each virulence-related gene contained a typical promoter containing-35 and-10 regions (Figure 4). The Fur binding site was also searched and predicted near the-35 and-10 regions. These putative Fur binding boxes of *Vshppd*, *Vsm,* and *Vsflic* showed 52.63, 63.16, and 52.63% homology to the consensus Fur binding site sequence (5′-GATAATGATAATGATTATC-3′), respectively (Figure 4D). This sequence alignment suggests that Fur might bind directly to the promoter and regulate the expression of *Vshppd*, *Vsm,* and *Vsflic*.

### Mutant of *Vibrio splendidus* showed lower pathogenicity in *Apostichopus japonicus*

**Figure 5:**
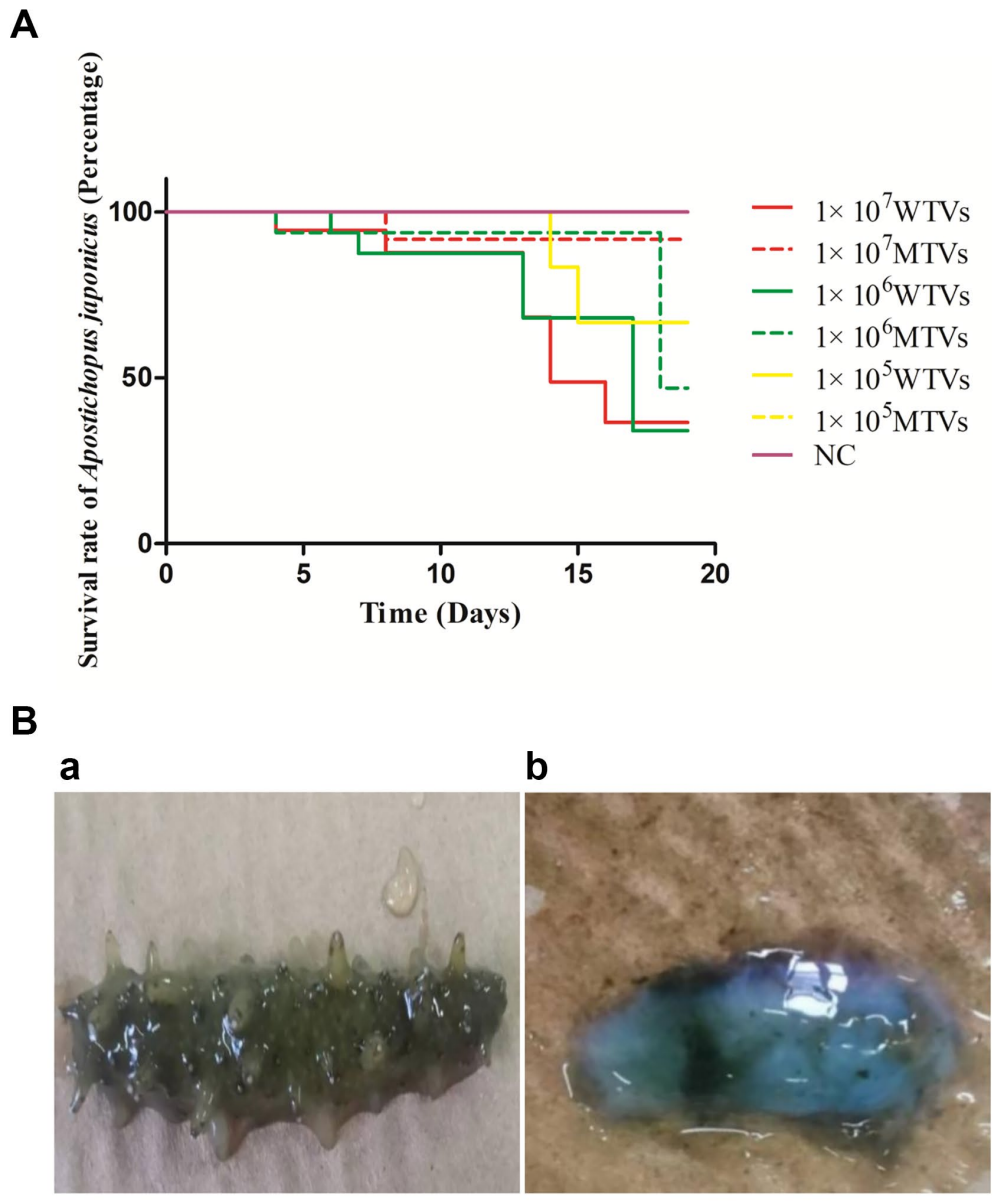
**(A)** The lethality of WTVs and MTVs. *Apostichopus japonicus* was randomly divided into seven tanks with 15 individuals each. The WTVs and MTVs strains used for infection were cultured in 2216E medium (24 h, 28°C) until OD_600_ was approximately 1.0. The strains were then washed and re-suspended in PBS (28°C). For survival assays, weight-matched *A. japonicus* individuals were infected with 1 × 10^7^, 1 × 10^6^, and 1 × 10^5^ CFU·ml^−1^ *V. splendidus* (WTVs or MTVs). *Apostichopus japonicus* infected with PBS was used as the negative control. The water temperature during infection was 16°C. The daily mortality of infected *A. japonicus* was recorded. **(B)** The observed symptoms of **(a)** MTVs and **(b)** WTVs. Dead *A. japonicus* were removed in a timely manner and photographed to observe symptoms.

In the artificial infection experiment, WTVs showed stronger pathogenicity than MTVs. Compared with the WTVs group, the *A. japonicus* infected with the MTVs strain showed a decrease in mortality and an obvious delay in the time of death (Figure 5A). The first mortalities of WTVs were observed 4 days post-infection with 1 × 10^7^ CFU·ml^−1^ WTVs, and the first mortalities of MTVs were observed 4 days post-infection with 1 × 10^6^ CFU·ml^−1^ MTVs. The total number of deaths in the WTVs group in 18 days were 7, 6, and 2 post-infection with 1 × 10^7^, 1 × 10^6^, and 1 × 10^5^ CFU·ml^−1^ WTVs, respectively. The total number of deaths in the MTVs group in 18 days were 1, 2, and 0 post-infection with 1 × 10^7^, 1 × 10^6^, and 1 × 10^5^ CFU·ml^−1^ MTVs, respectively. The *A. japonicus* infected with WTVs showed obvious characteristics of SUS, such as head shaking and skin ulceration. However, the *A. japonicus* group infected with MTVs did not show obvious characteristics of SUS (Figure 5B). No death occurred in the negative control group during the whole experiment. The median lethal doses (LD_50_) of WTVs and MTVs calculated by SPSS were 9.116 × 10^6^ and 1.658 × 10^11^ CFU·ml^−1^, respectively. These results indicate that *Vsfur* is crucial to the pathogenicity of *V. splendidus*.

### Mutant of *Vibrio splendidus* showed lower colonization ability compared to WTVs

**Figure 6:**
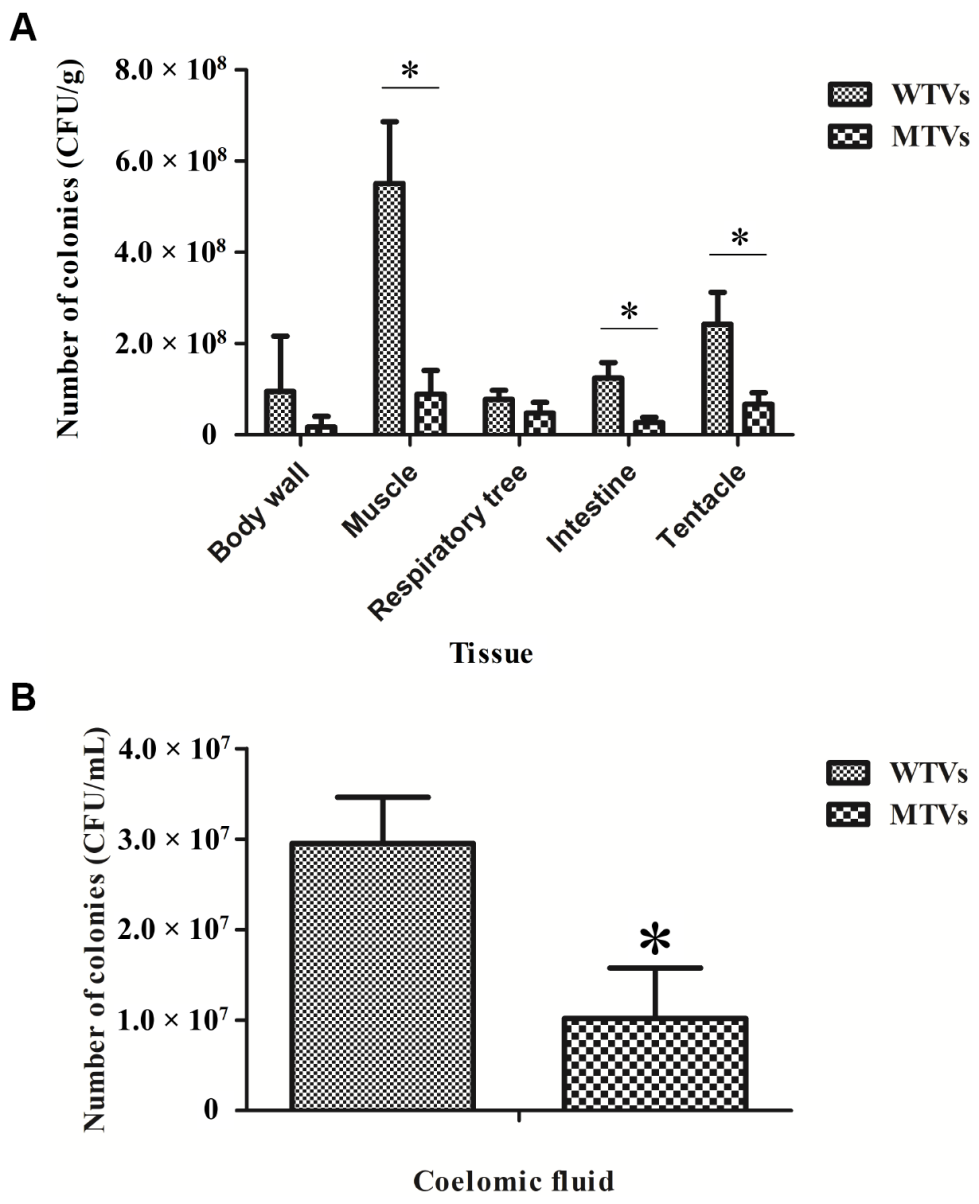
The colonization abilities of WTVs and MTVs to different *A. japonicus* tissues **(A)** and coelomic fluids **(B)** were demonstrated by colony counting. A. japonicus was soaked in WTVs and MTVs (1.0 × 10^7^ CFU·ml^−1^) for 48 h infection. The *A. japonicus* tissues were weighed and homogenized. The coelomic fluids were filtered through a 200-mesh. The homogenized solution was diluted in gradients and then coated on 2216E plates.

The quantitative detection of cell colonization in various tissues of *A. japonicus* after immersion infection with WTVs and MTVs is shown in Figure 6. The colonization quantities of WTVs to the body wall, muscle, respiratory tree, intestine, and tentacle tissues were 9.53 × 10^7^, 5.51 × 10^8^, 7.77 × 10^7^, 1.24 × 10^8^, and 2.42 × 10^8^ CFU·g^−1^, respectively. The colonization amount of WTVs to the coelomic fluid was 2.95 × 10^7^ CFU·ml^−1^. The colonization quantities of MTVs to all tissues except the body wall and respiratory tree decreased. The colonization quantities of MTVs to the body wall, muscle, respiratory tree, intestine, and tentacle tissues were 1.71 × 10^7^, 8.92 × 10^7^, 4.78 × 10^7^, 2.70 × 10^7^, and 6.71 × 10^7^ CFU·g^−1^, respectively. The colonization amount of MTVs to the coelomic fluid was 1.02 × 10^7^ CFU·ml^−1^.

### Mutant of *Vibrio splendidus* showed lower swarming motility to WTVs

**Figure 7:**
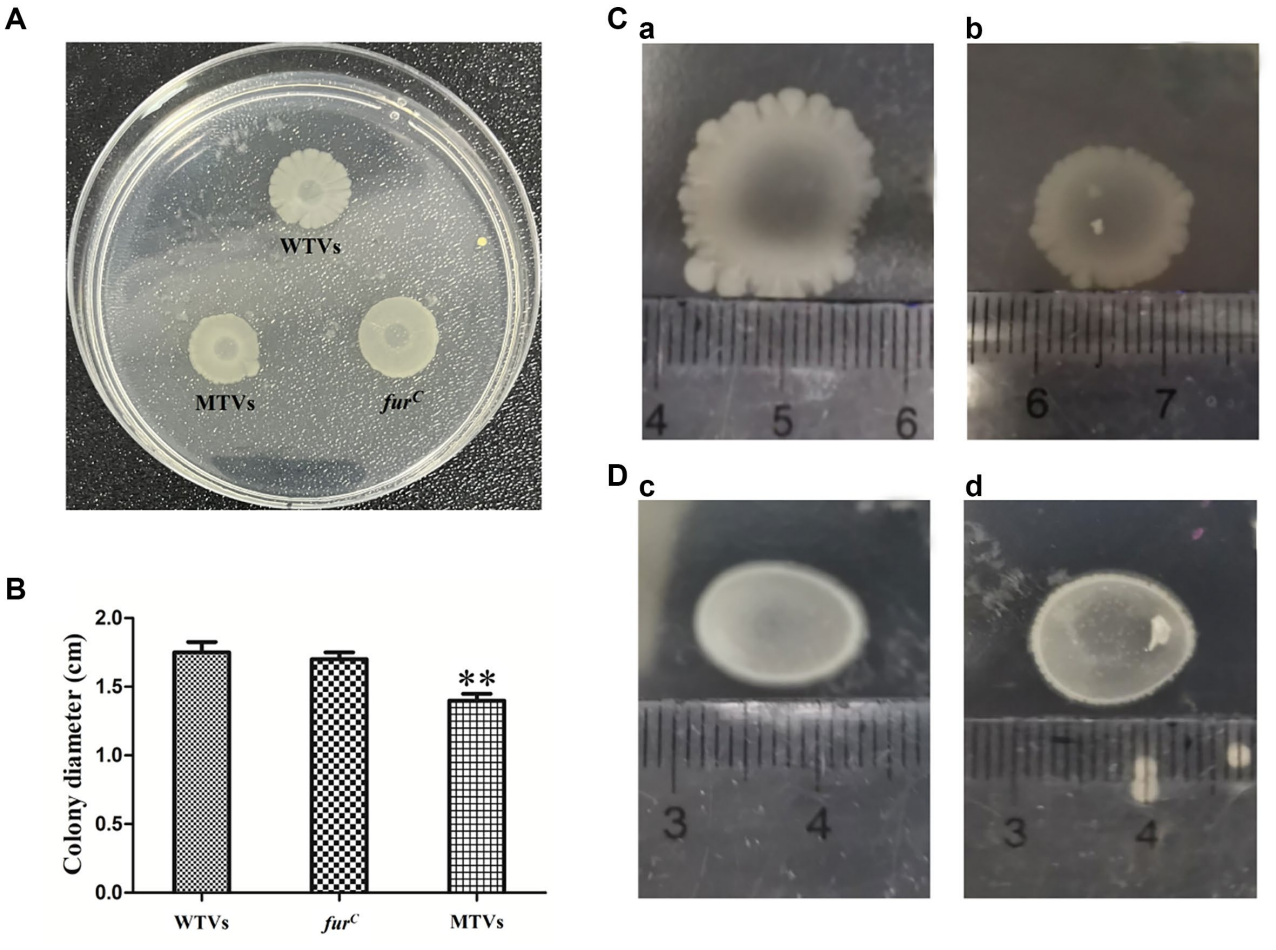
**(A)** Swarming motility of WTVs, MTVs, and *fur^C^* in normal conditions. **(B)** Bar graph of the swimming motility of WTVs, MTVs, and *fur^C^*. **(C)** The same concentration of WTVs **(a)** and MTVs **(b)** (2 μl) were dropped on low-agar 2216E plates supplemented with 100 μM FeCl_3_ and cultured for 48 h at 28°C. **(D)** The same concentration of WTVs **(c)** and MTVs **(d)** (2 μl) was dropped on low-agar 2216E plates supplemented with 200 μM DP and cultured for 48 h at 28°C. The error line represents the SD (*n* = 5). Asterisks represent the significant difference (*p<0.05, **p<0.01).

The motility of WTVs, MTVs, and *fur^C^* strains was measured in 2216E agar plates, and the results demonstrated an obvious difference in the swarming motility of WTVs and MTVs (Figure 7A). The swarming halo diameter of WTVs was 3–4 mm more than that of MTVs at each time point and the diameter of MTVs was 79–81% (*p* < 0.01) of that of WTVs in normal conditions (Figure 7B). Meanwhile, the swarming motility of MTVs was reduced by approximately 0.68-fold compared with that of WTVs in iron-replete conditions (Figure 7C). Under the iron-starved conditions, the motility of WTVs and MTVs was restricted and could not be spread normally (Figure 7D). These results suggest that *Vsfur* contributes to the swarming motility of *V. splendidus*.

### Mutant of *Vibrio splendidus* showed lower biofilm formation to WTVs

**Figure 8:**
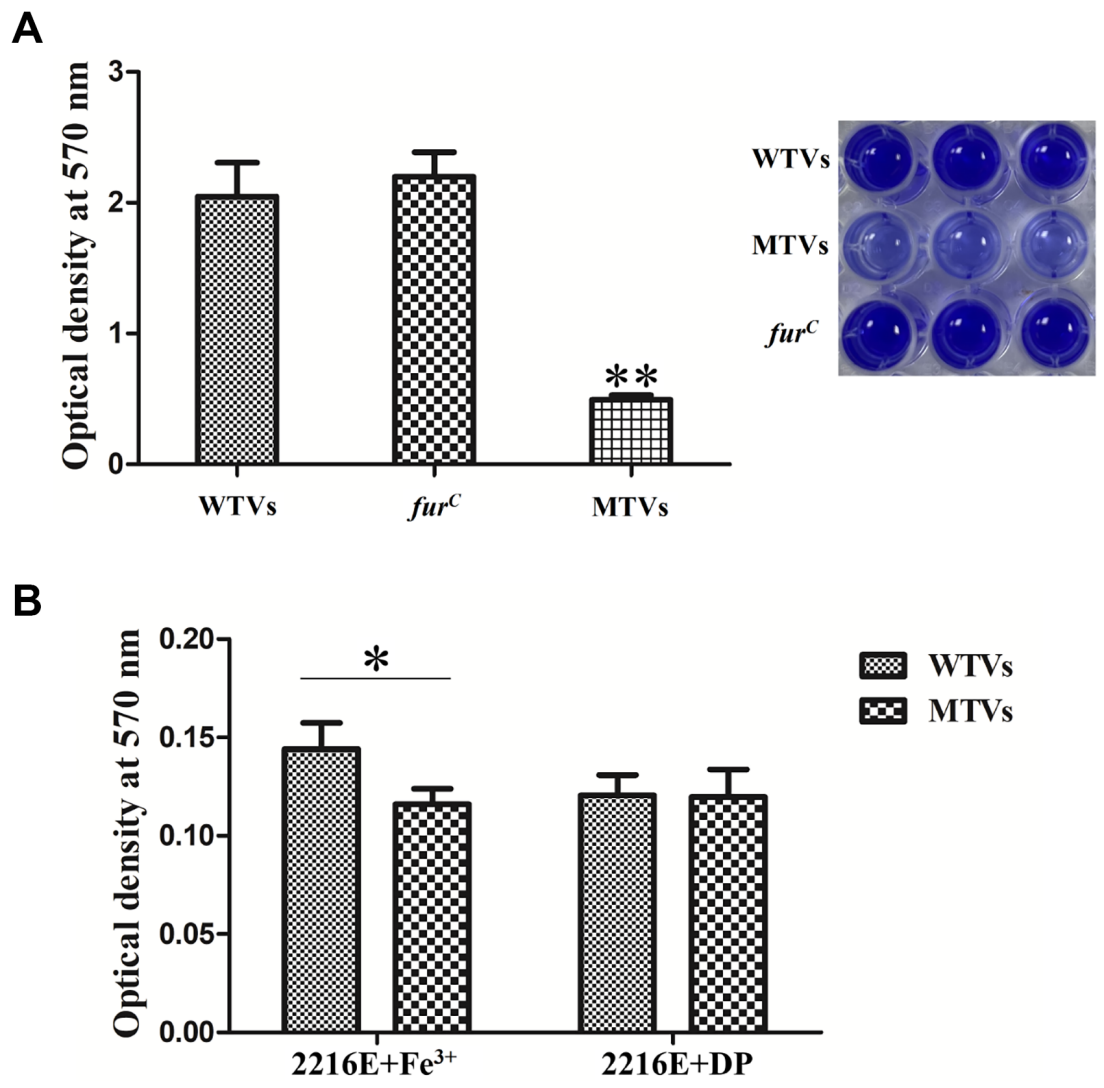
**(A)** Biofilm formation of WTVs, MTVs, and *fur^C^* in normal conditions. **(B)** Biofilm formation of WTVs and MTVs in iron-replete conditions and iron-starved conditions. The error line represents the SD (*n* = 5). Asterisks represent the significant difference (*p < 0.05, **p < 0.01).

The biofilm formation was determined using a crystal violet (CV) staining assay. The biofilms of WTVs, MTVs, and *fur^C^* that attached to 96-well plates were analyzed in normal 2216E medium. Consistent with the results of swarming motility, the biofilm formation of MTVs on the surface of a 96-well plate was 24% (*p* < 0.01) and 22% of WTVs and *fur^C^*, respectively (Figure 8A). The biofilm formation of MTVs was 81% (*p* < 0.05) of WTVs in iron-replete conditions. Under the iron-starved conditions, the biofilm formation of WTVs and MTVs showed no significant difference (Figure 8B). Taken together, these results indicate that deletion of *Vsfur* results in decreased biofilm formation, which depends on the presence of Fe^3+^.

## Discussion

Many genes have been proven to be involved in regulating the virulence of aquatic pathogenic bacteria by knock-out technology (36, 37). Differently from other bacteria, attempts to obtain *fur* deletion mutants of *V. splendidus* failed, leading to the assumption that Fur is an essential protein in *V. splendidus*. While several attempts to obtain *Vsfur* in-frame deletion mutants by suicide vector failed, we constructed a *Vsfur* knock-down mutant of *V. splendidus* through antisense RNA interference. The recombinant plasmid pET28aVsfur containing promoter P_Trc_ could efficiently express the interference sequence in *V. splendidus* (28, 35). In previous studies, *fur* mutants of other *Pseudomonas aeruginosa* and *Acidovorax citrulli* have shown significantly reduced growth rates in low-and high-iron environments (18, 38). In this study, the growth rate of the MTVs strain was the same as that of the WTVs strain in iron normal conditions.

Bacteria depend on Fur to regulate the absorption and utilization of iron, which has a critical impact on bacterial survival and virulence (39). Moreover, recent studies have demonstrated that Fur can function as both an activator and a repressor on a global level through both direct and indirect mechanisms to affect a variety of virulence-related traits (14). However, no studies on the contribution of the knock-out of the *fur* gene to the pathogenesis of *V. splendidus* have been reported. In this study, the expression of the *Vsfur* mRNA level was not significantly different in MTVs at the OD_600_ of 0.6 compared with the WTVs. After that, the expression level was significantly downregulated and maintained a low level at the OD_600_ of 1.0 and 1.5. It might be that the interference had not occurred effectively in the early log phase of bacteria. With the continuous copy of the vector, a large number of interfering sequences were expressed, leading to the effective blocking of the *Vsfur* gene. Meanwhile, the result showed that the expression of virulence-related genes *Vshppd* and *Vsm* upregulated significantly with the decrease of the *Vsfur* gene. We speculate that it was the high-iron concentration inhibition phenomenon dependent on Fur. When the concentration of iron reaches a stable state, Fur binds and forms a complex with Fe^2+^, and the resulting Fur-Fe^2+^ complex binds to the promoter region of the target gene (40). This binding results in the transcriptional repression of genes involved in iron acquisition and storage (21). The siderophore synthesis pathway of *E. coli*, *Vibrio cholerae,* and *Bacillus subtilis* showed a high-iron concentration inhibition mode (23, 41). In addition to iron metabolism-related genes, the expression of the hemolysin synthesis gene *hly* (42), the outer membrane protein synthesis gene *irgA* of *V. cholerae* (43), and the catalase gene *catC* of *Streptomyces coelicolor* (44) was inhibited by Fur. Our previous research demonstrated that HGA-melanin produced by *Vshppd* with the ability to reduce Fe^3+^ to Fe^2+^, contributes to the ferrous iron uptake pathway in *V. splendidus* (45). In addition, BPROM prediction suggested that the upstream of the *Vshppd* and *Vsm* had apparent Fur binding sites. Overall, these results demonstrated that Fur may regulate the expression of *Vshppd and Vsm* through a high-iron concentration inhibition mode in *V. splendidus*. However, the detailed regulation mechanism of Fur needs to be investigated in future studies.

Motility is an important virulence determinant during the adhesion and invasion stages in many pathogenic bacteria and enables bacterial cells to swim to nutrient-rich niches or avoid environmental stresses (46). Previous research has suggested that Fur directly activates the transcription of toxin coregulated pilus *tcpA,* which was necessary for colonization by binding to their promoters in *V. cholerae* (47). In *Shigella flexneri*, Fur could indirectly activate the *icsA* gene that facilitates bacterial movement by activating VirF (48). Our results demonstrate that the swarming motility of the MTVs was significantly reduced compared to the WTVs. Consistent with these findings, gene expression levels of *Vsflic* were significantly reduced in the MTVs. Our previous research demonstrated that FliC could contribute to swarming motility in *V. splendidus* (49). Combining these results, Fur could positively regulate the expression of *Vsflic* to contribute to swarming motility in *V. splendidus,* whereas the repressive mechanism of Fur has been well understood; emerging research on direct and indirect Fur-mediated activation mechanisms will reveal novel global regulatory circuits (22).

In consideration of the complex interaction relationship between motility and biofilm formation (50), we further compared the biofilm formation of MTVs and WTVs at different iron concentrations. The results showed that the biofilm formation of MTVs was decreased compared to the WTVs in normal and iron-replete conditions. Considering that low-iron concentration limits the growth of bacteria, the biofilm formation of WTVs and MTVs showed no significant difference. Moreover, despite the increased expression of virulence-related genes in MTVs, motility and adhesion are prerequisites for pathogen infection (51). We speculated that the pathogenicity of MTVs in *A. japonicus* was lower than that of WTVs. The results confirmed that *A. japonicus* infected with the MTVs strain exhibited a significant reduction in mortality, a remarkable delay in the time of death, and alleviated SUS symptoms compared with the WTVs group. All these findings support that the knock-down of *Vsfur* leads to the decrease of swarming motility and biofilm formation in *Vibrio splendidus*, affecting its virulence in *Apostichopus japonicus*.

In summary, the knock-down of the *Vsfur* gene in *V. splendidus* showed no effect on growth, but MTVs infection resulted in a reduced death rate, delayed time of death, and alleviated SUS symptoms in *A. japonicus*. Vsfur regulates biofilm formation and swarming motility, which are responsible for the virulence of *V. splendidus*. These findings will provide a theoretical basis for the prevention and control of SUS.

## Data availability statement

The original contributions presented in the study are included in the article/supplementary material, further inquiries can be directed to the corresponding author.

## Ethics statement

The animal study was reviewed and approved by the Experimental Animal Ethics Committee of Ningbo University, China. Written informed consent was obtained from the owners for the participation of their animals in this study.

## Author contributions

YS performed the experiments and interpreted the data. CYL, FD, and YZ performed the partial experiment. CHL contributed new reagents, analytic tools, and revised the manuscript. WL participated in the experimental design, interpreted the data, and wrote the manuscript. All authors read and approved the manuscript.

## Funding

This work was financially supported by the National Natural Science Foundation of China (32102843 and 32073003), Zhejiang Provincial Natural Science Foundation (LY23C190003), and the K. C. Wong Magna Fund at Ningbo University.

## Conflict of interest

The authors declare that the research was conducted in the absence of any commercial or financial relationships that could be construed as a potential conflict of interest.

## Publisher’s note

All claims expressed in this article are solely those of the authors and do not necessarily represent those of their affiliated organizations, or those of the publisher, the editors and the reviewers. Any product that may be evaluated in this article, or claim that may be made by its manufacturer, is not guaranteed or endorsed by the publisher.

## Footnotes

^1^ http://www.softberry.com
